# An indirect debiasing method: Priming a target attribute reduces judgmental biases in likelihood estimations

**DOI:** 10.1371/journal.pone.0212609

**Published:** 2019-03-07

**Authors:** Kelly Kiyeon Lee

**Affiliations:** Department of Marketing, McDonough School of Business, Georgetown University, Washington, District of Columbia, United States of America; Loughborough University, UNITED KINGDOM

## Abstract

Understanding the underlying psychological process that leads to a bias is crucial for developing remedies to correct or reduce the bias. As one of the psychological processes that underlie judgmental biases, attribute substitution provides an explanation as to why people rely on heuristics and commit judgmental biases. Attribute substitution occurs when people make a judgment that requires the use of a target attribute, but make the judgment using a heuristic attribute that comes more readily to mind. This substitution inevitably introduces systematic errors because these two attributes are different. The current work explores an indirect debiasing method—the priming of a target attribute. Across three experiments, we demonstrate that priming a target attribute in prior tasks reduces judgmental biases in likelihood estimations: ratio-bias and base-rate neglect. However, this outcome only occurs when participants have enough cognitive resources. When they experience cognitive load, the priming of the target attribute does not reduce their judgmental biases.

## Introduction

People are subject to biases when making probability judgments. Since the classic article by Tversky and Kahneman [1], a substantial literature has examined how these biases may be corrected. Endeavors to correct or reduce biases are referred to as debiasing [2]. In early work on debiasing, Fischhoff [3] provides a review of various debiasing methods designed to reduce two specific biases: hindsight bias and overconfidence. This work divides debiasing methods according to whether responsibility for the biases is on the decision-maker, the task, or some mismatch between the two, and provides different strategies for developing debiasing techniques.

To build a more comprehensive understanding about biases and debiasing methods, Arkes [4] proposed that understanding the causes of different types of biases promotes identifying effective debiasing techniques, and categorized biases into three types of biases—psychophysically-based errors, association-based errors, and strategy-based errors. *Psychophysically-based errors* occur when people map physical stimuli onto psychological responses in a nonlinear manner. The typical examples of this type of biases are reference point effects related to prospect theory [5, 6]. Adding new gains or losses, changing one’s reference point, and reframing losses as gains are shown to be effective in reducing psychophysically-based errors [7–9]. *Association-based-errors* involve using associations within semantic memory that are irrelevant or counterproductive on the judgment or decision. For example, people who imagined experiencing certain events evaluated those events as more likely to occur than those who did not imagine those events. The activity of imagining can make events more available in long-term memory, which led people to judge those events to be more probable [10]. Making people “consider the opposite” and merely cuing a debiasing behavior rather than explicitly instructing people in a different judgment behavior are considered to be effective as debiasing strategies in this category [11, 12]. *Strategy-based errors* arise when people employ a suboptimal strategy rather than an optimal strategy. Using the suboptimal strategy is beneficial because it is fast and easy to execute. Although the suboptimal strategy may be adaptive, it can be costly and result in more errors. One possible solution to improve judgment in this category, is to raise the cost of using the suboptimal strategy [13, 14].

Larrick [15] linked the three different types of biases proposed by Arkes [4] to two cognitive systems—System 1 (intuition) and System 2 (reasoning). The processes of System 1 are automatic, effortless, and fast whereas the processes of System 2 are controlled, effortful, and slow [16, 17]. Larrick [15] argued that psychophysically-based errors and association-based errors are attributable to System 1 processes, and strategy-based errors are attributable to System 2 processes.

Decision biases are not only limited to cognition, but also are rooted in motivation. In particular, Montibeller and von Winterfeldt [2] suggested that individuals exhibit motivational biases because they can be influenced by the desirability of decision outcomes. For example, experts may provide overly optimistic estimates for a preferred action.

In this investigation, we focus on association-based errors and develop an indirect debiasing method drawing on the Model of Heuristic Judgment proposed by Kahneman and Frederick [16, 17]. Contrary to direct debiasing techniques which are executed by altering the decision maker in an explicit way (e.g., “consider the opposite” strategy or cuing a debiasing behavior), indirect debiasing techniques can be developed by altering the environment without requiring the decision maker’s awareness of the underlying process why the bias occurs [15, 18]. According to the Model of Heuristic Judgment [16, 17], there are two different systems related to the decision-maker’s judgment—System 1 (intuition) and System 2 (reasoning). The processes associated with System 1 are automatic, effortless, fast, parallel, and slow-learning whereas the processes associated with System 2 are controlled, effortful, slow, serial, and flexible. Association-based biases occur because individuals substitute the heuristic attribute, which is more readily accessible yet irrelevant, for the target attribute, which is relevant and should be used to make a correct judgment. Kahneman and Frederick [16] propose three conditions under which attribute substitution occurs: (1) the target attribute is relatively inaccessible; (2) the heuristic attribute is highly accessible; (3) System 2 (reasoning) fails to reject the substitution of the heuristic attribute.

Drawing on this model, we introduce a novel indirect debiasing technique—the priming of the target attribute. As discovered decades ago, priming of semantic concepts increases their accessibility [19]. Priming has been shown to affect judgments in a variety of domains such as affective evaluations, persuasion, semantic effects, social stereotypes, and behavior [20–24]. We used priming because the likelihood of making a correct judgment is dependent on the accessibility of the target attribute, and does not require conscious awareness. To overcome decision biases, Kenyon and Beaulac [25] proposed four different levels of debiasing methods depending on the extent to which the sources of biases are internal or external. We predict that priming the target attribute increases the accessibility of the target attribute in the decision maker’s memory, which in turn reduces the likelihood of making the judgmental bias or error.

In addition, we examine whether individuals need cognitive resources to reduce the bias That is, we explore whether using a target attribute as a relevant source of information for making decisions requires cognitive resources. According to Kahneman and Frederick [16], there is a sequence involved in judgmental processes whereby System 1 (intuition) precedes System 2 (reasoning). Specifically, Kahneman and Frederick [16] wrote “System 1 quickly proposes intuitive answers to judgment problems as they arise, and System 2 monitors the quality of these proposals, which it may endorse, correct, or override” (p 51).

Prior work suggests that errors commonly occur even though System 2 monitors the quality of both mental operations and overt behavior [26, 27]. Kahneman and his colleagues explain that many intuitive erroneous judgments are expressed because the self-monitoring function is normally quite lax and effortful [16, 28].

In order to test whether cognitive resources are required to reduce judgmental biases, when the target attribute is made more accessible by the indirect priming method, we used a cognitive load manipulation. If the process of making the correct judgments requires cognitive resources, a high cognitive load will impair this process as opposed to a low cognitive load. Consequently, the likelihood of correcting judgments may decrease with the high cognitive load.

We conducted three experiments to test whether the priming of a target attribute reduces two judgmental biases in likelihood estimations: ratio-bias and base-rate neglect.

## Experiment 1

Experiment 1 examines whether priming the target attribute reduces judgmental errors in the Jelly Beans task [29]. This task shows *a ratio-bias phenomenon* which refers to “the perception of the likelihood of a low-probability event as greater when it is presented in the form of larger rather than smaller numbers” [30]. For instance, people prefer to draw from a tray with “larger” number of beans (e.g., 10 red out of 100 jelly beans) over a tray with “smaller” number of beans (e.g., 1 red out of 10 jelly beans) hoping of obtaining a winning red jelly even though the ratios of winning between the two trays are identical. The ratio-bias phenomenon is known to be robust in various domains such as self-reported gambling in real life [31], heuristic responses to vignettes [31], depression [32], and even health [33]. This phenomenon is attributed to a tendency to focus on the frequency of the numerator (i.e., heuristic attribute) instead of the overall probability (i.e., target attribute). Selecting one of two trays that offer equal probabilities is, in itself, not a judgmental error. However, the preference for a 9% over a 10% probability does reflect an error in judgment [31].

We predicted that increasing the accessibility of the target attribute through priming results in less bias because there will be less substitution of the heuristic attribute for the target attribute.

### Materials and methods

This research (Experiments 1–3) was approved by the Research Ethics Board at the University of Toronto (Approval Number: # 22120). In Experiment 1, eighty-four business undergraduate students at a North American university were randomly assigned to one of the two priming conditions: no priming vs. target attribute priming. Upon arrival, participants were informed that they would participate in two ostensibly unrelated studies. The first study was a visual detection task which was designed to prime half of participants with the target attribute, and the other half with no words [22]. Participants were told that they would participate in the visual detection task and their task was to identify whether each string of letters presented on a computer screen contained two vowels (press “Z” key) or not (press “M” key). They were told that the researchers were interested in how quickly university students responded to visual stimuli. Participants first completed three practice trials where they saw three strings of letters (e.g., mecedjz). Next, they started the main task. In this task, participants in the target attribute priming condition were first primed (20 ms) with five words before they saw a string of letters (e.g., qjxriadpl, tkfkdirgo). The five words related to the target attribute (i.e., probability, proportion, ratio, likelihood, and odds) were shown twice in a random order. In the no-priming condition, no words were primed prior to the ten strings of letters. In both conditions, when participants saw the string of letters, they indicated whether it contained two vowels or not.

After all participants finished the first study, they were asked to move to a desk which was located on the opposite side of the cubicles in the lab. Two trays for the jelly beans task were arranged on this desk. First, participants were given a brief paragraph to read. The vignette for the jelly beans task was as follows:

As you can see, there are two trays on the table. One tray (Tray A) contains 10 jelly beans (1 red bean and 9 white beans) while the other tray (Tray B) contains 100 jelly beans (9 red beans and 91 white beans). If you draw a red jelly bean, you can participate in a lottery in which the winner will receive $50. However, if you draw a white jelly bean, you will win nothing.

Given this information, participants were told that they had a real opportunity to draw one jelly bean from one of the two trays for a real lottery. Before the actual drawing, they were asked to choose which tray they wanted to draw from. Those who draw a red bean were given a lottery ticket in which they wrote their name and contact information for the drawing. Written and signed informed consent was obtained from participants in Experiments 1–3.

### Results and discussion

**Fig 1:**
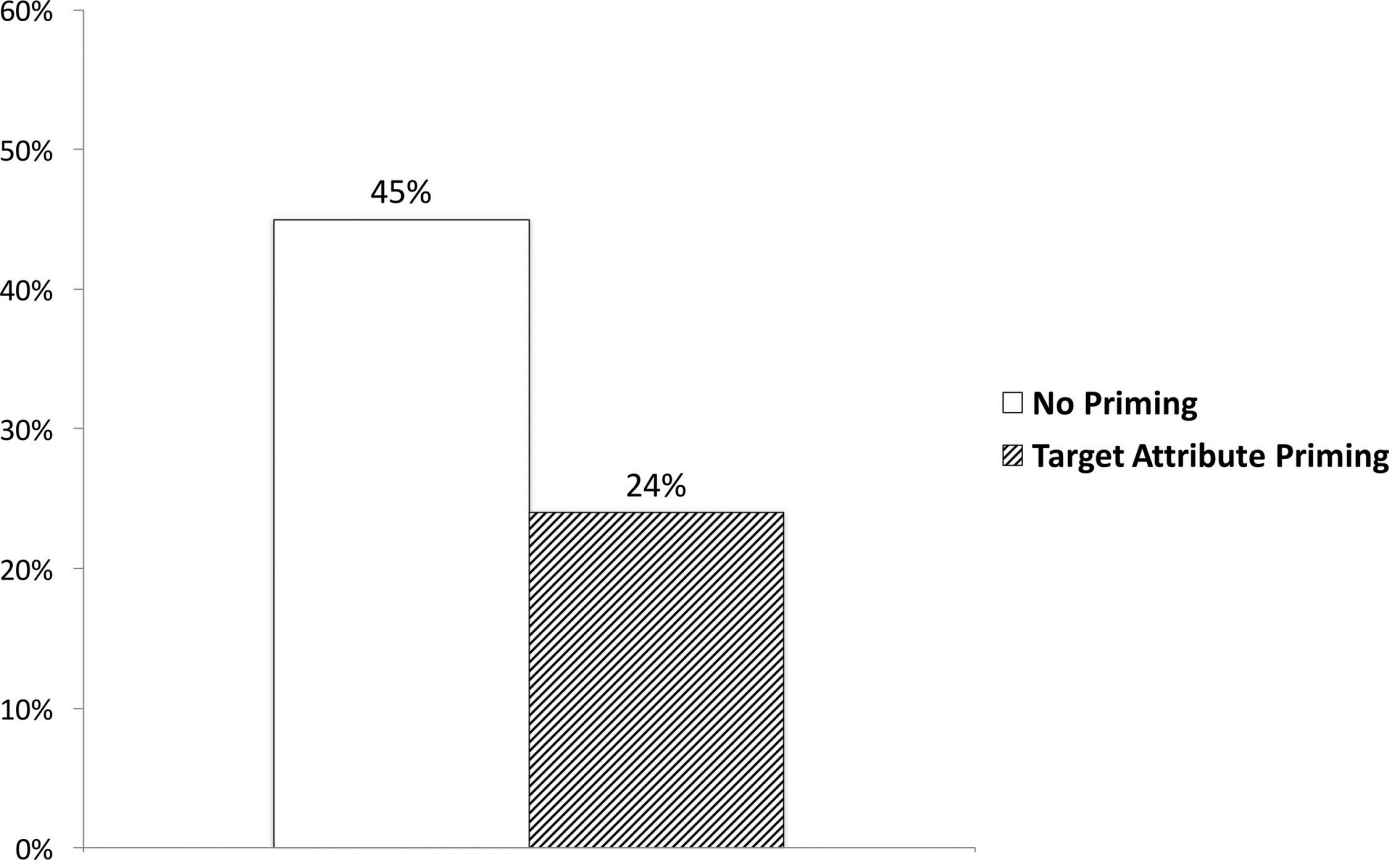
The effect of priming on the ratio-bias in Experiment 1. Higher percentages indicate greater ratio-bias (choosing the 9% tray over the 10% tray).

Since choosing the 9% tray indicated the ratio-bias, we report the percentage choosing the 9% tray. An overall chi-square test revealed that participants primed with the target attribute (24%) chose the 9% tray significantly less often than those who were not primed (45%): *χ*^2^(*df* = 1, N = 84) = 4.27, *p* = .039, Φ = -.23 (Fig 1). This result suggests that exposure to words related to the target attribute decreased the likelihood of making a judgmental error even though participants were not aware of why this occurred.

## Experiment 2

In Experiment 1, we show that increasing the accessibility of the target attribute through priming reduces ratio-bias. In Experiment 2, we also primed the heuristic attribute to examine whether it increases the ratio-bias. Since the Model of Heuristic Judgment proposes that the heuristic attribute is highly accessible when making the judgment, priming the heuristic attribute should not increase its accessibility, so this should not increase the ratio-bias.

### Materials and methods

Ninety-two business undergraduate students at a North American university were randomly assigned to one of the three priming conditions: no priming, target attribute priming, and heuristic attribute priming.

Participants first completed the same visual detection task as in Experiment 1. In the target attribute priming condition, the same five words (i.e., probability, proportion, ratio, likelihood, and odds) were primed twice in a random order. In the no priming condition, no words were primed. In the heuristic attribute priming condition, five words related to the heuristic attribute (i.e., frequency, number, many, more, and numerous) were shown twice in a random order. In all three conditions, when participants saw the string of letters, they indicated whether it contained two vowels or not.

Lastly, participants were asked to calculate the probability for a sample question to check whether participants have correct knowledge of probability: “At Kennedy Middle School, 3 out of 5 students make the honor roll. What is the probability that a student makes honor roll?”. According to Camerer and Hogarth [34], having knowledge or “cognitive capital” about a correct judgment is crucial for improving performance. In the Jelly Beans task, it is important for participants to have knowledge of probability to make a correct judgment.

### Results and discussion

**Fig 2:**
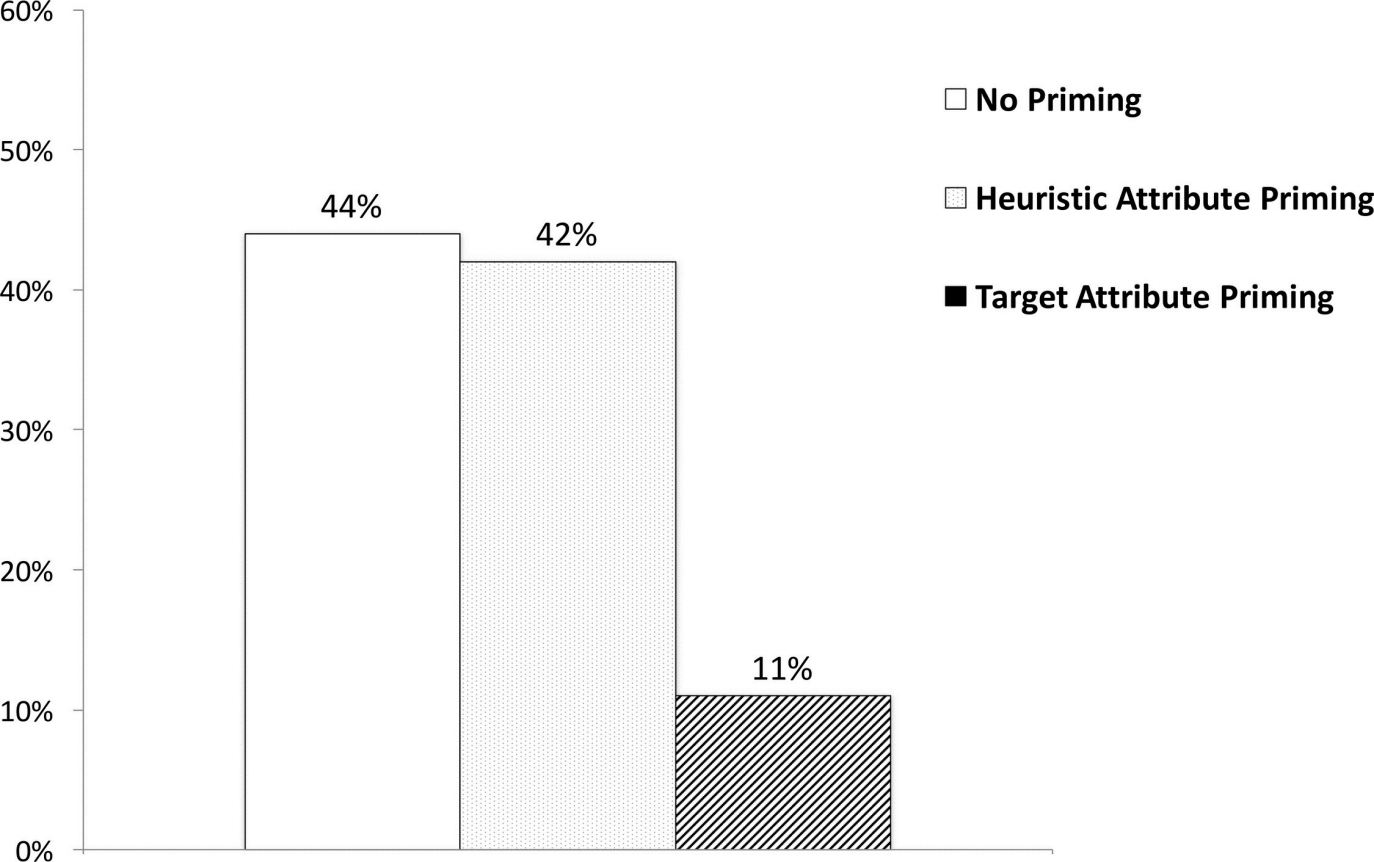
The effect of priming on the ratio-bias in Experiment 2. Higher percentages indicate greater ratio-bias (choosing the 9% tray over the 10% tray).

In our initial analyses, only 83 participants were included after excluding 9 participants who did not correctly answer the sample probability question at the end of the experiment. An overall chi-square test revealed a significant effect of priming (*χ*^2^(*df* = 2, *N* = 83) = 8.39, *p* = .015, Φ = .32; see Fig 2). Participants in the target attribute priming condition (11%) chose the 9% tray significantly less often than participants in the no priming condition (44%; *χ*^2^(*df* = 1, *N* = 59) = 7.61, *p* = .006, Φ = -.36) and the heuristic attribute priming condition (42%; *χ*^2^(*df* = 1, *N* = 51) = 6.25, *p* = .012, Φ = .35). The percentage of participants selecting the 9% tray were virtually identical in the no priming and the heuristic attribute priming condition (*χ*^2^(*df* = 1, *N* = 56) = .02, *p* = .876, Φ = -.02), which indicates that priming the heuristic attribute does not increase the ratio-bias.

We conducted the same analysis by including 9 participants who failed to correctly answer the sample probability question, and we found a similar pattern of results: An overall chi-square test revealed a significant main effect of priming on the tray choice (*χ*^2^(*df* = 2, *N* = 92) = 7.22, *p* = .027, Φ = .28). Specifically, the percentage of choosing the 9% tray in the target attribute priming condition (16%) was significantly lower than that in the no priming condition (44%; *χ*^2^(*df* = 1, *N* = 68) = 6.59, *p* = .010, Φ = -.31) and that in the heuristic attribute priming condition (42%; *χ*^2^(*df* = 1, *N* = 56) = 4.74, *p* = .029, Φ = .29). However, there was no significant difference between the heuristic attribute priming condition (42%) and the no priming condition (44%; *χ*^2^(*df* = 1, *N* = 60) = .05, *p* = .832, Φ = -.03) in terms of choosing the 9% tray.

The findings of Experiment 2 provide further evidence for our predictions by replicating the finding that priming the target attribute reduces the ratio-bias. Importantly, we also show that the heuristic attribute is readily accessible when making a judgment and that priming the heuristic attribute does not increase the ratio-bias.

## Experiment 3

The purpose of Experiment 3 is to generalize the findings by using a different judgmental bias (i.e., base-rate neglect) and to test whether the process of reducing judgmental biases requires cognitive resources. In Experiment 3, the problem-solving task used the professor-and-non-professor problem [35], which is an analogue of the engineer-and-lawyer problem [36]. The engineer-and-lawyer problem illustrates a fallacy which reflects the use of the representativeness heuristic. For example, in the engineer-and-lawyer problem, participants are told that the percentage of engineers in a given sample is very low (e.g., 30%). They are then given a description of an individual which is similar to the stereotype of engineers (e.g., having no interest in political and social issue and spending most of his time in home carpentry, sailing, and mathematical puzzles). The participants’ estimation of the probability that a person is an engineer is much higher (e.g., 80%) than the base rate (e.g., 30%) because people tend to rely on the description of the person rather than the base rate. Base-rate neglect in probability judgments is problematic because people violate the fundamental Bayesian rule of statistical prediction. Although some find that providing the problem in frequency format [37–39] or showing natural sampling [35] can help to reduce base-rate neglect, to the best of our knowledge, our research is the first to examine whether an indirect debiasing method (i.e., priming the target attribute) will reduce base-rate neglect.

To test whether cognitive resources are also required to reduce the bias with the increased accessibility of the target attribute, we manipulated cognitive load. In Experiments 1 and 2, increasing the accessibility of the target attribute reduced the bias when participants could use all their cognitive resources to make the judgment. However, these findings do not explain whether individuals need cognitive resources to reduce the bias. If individuals are still able to reduce the bias under a high cognitive load, it would indicate that increased accessibility of the target attribute is sufficient to reduce the bias. However, if individuals are less able to reduce the bias under a high cognitive load, it suggests that cognitive load impairs the process of reducing the bias.

### Materials and methods

One hundred fifteen business undergraduate students at a North American university were randomly assigned to a 2 (priming: no priming vs. target attribute priming) x 2 (cognitive load: low vs. high) between-subjects design. To manipulate cognitive load, we used a number rehearsal dual task which has been successfully used as a cognitive load manipulation [40–43].

Participants were informed that they would participate in two unrelated studies. In the first study, we used the same visual detection task as in Experiment 1 to prime the target attribute. After finishing the first study, participants were provided a questionnaire which included the professor-and-non-professor problem [35] with a modification of the base rate. They were told that the purpose of the study was to understand how university students answered various types of questions. Before being exposed to the professor-and-non-professor problem, participants were asked to memorize either a random 2- or 9-digit number. They then were asked to read the professor-and-non-professor problem in which they were first told that they would be provided with a description of an individual which was randomly selected from a sample which contained 17.6% professors and 82.4% non-professors. They were then given a description of a person (e.g., typical characteristics of a professor such as attending international conventions and wearing a suit and a tie). Their task was to rate the probability that the person is a professor given the description of the person and the base rate. After rating the probability, participants were asked to write down the number that they memorized. Next, they answered several unrelated filler questions. And then, they were asked to rate the difficulty of memorizing the number on a 9-point scale (1 = extremely easy vs. 9 = extremely difficult). Lastly, participants completed the PANAS scale [44] to examine whether our cognitive load manipulation influences participants’ mood, which in turn affects participants’ reliance on stereotyped thinking [45].

## Results and discussion

### Manipulation check

One hundred two participants were included in our analysis after excluding 13 participants who recalled four or less digits correctly in the high load conditions based on the a priori cutoff from previous literature [42]. However, if we include these 13 participants in our major analyses (manipulation check, probability, base-rate, and the PANAS scale), no differences in effects for analyses were observed. All of the participants in the low cognitive load conditions successfully reported the 2-digit number. In support of the cognitive resource manipulation, individuals who were asked to memorize the 9-digit number (*M*_*9-digit*_ = 3.56, *SD*_*9-digit*_ = 2.11) reported greater difficulty memorizing the test number than those who were given the 2-digit number (*M*_*2-digit*_ = 1.22, *SD*_*9-digit*_ = 0.50; *t*(100) = -7.90, *p* < .001, *d* = .67).

### Probability

**Fig 3:**
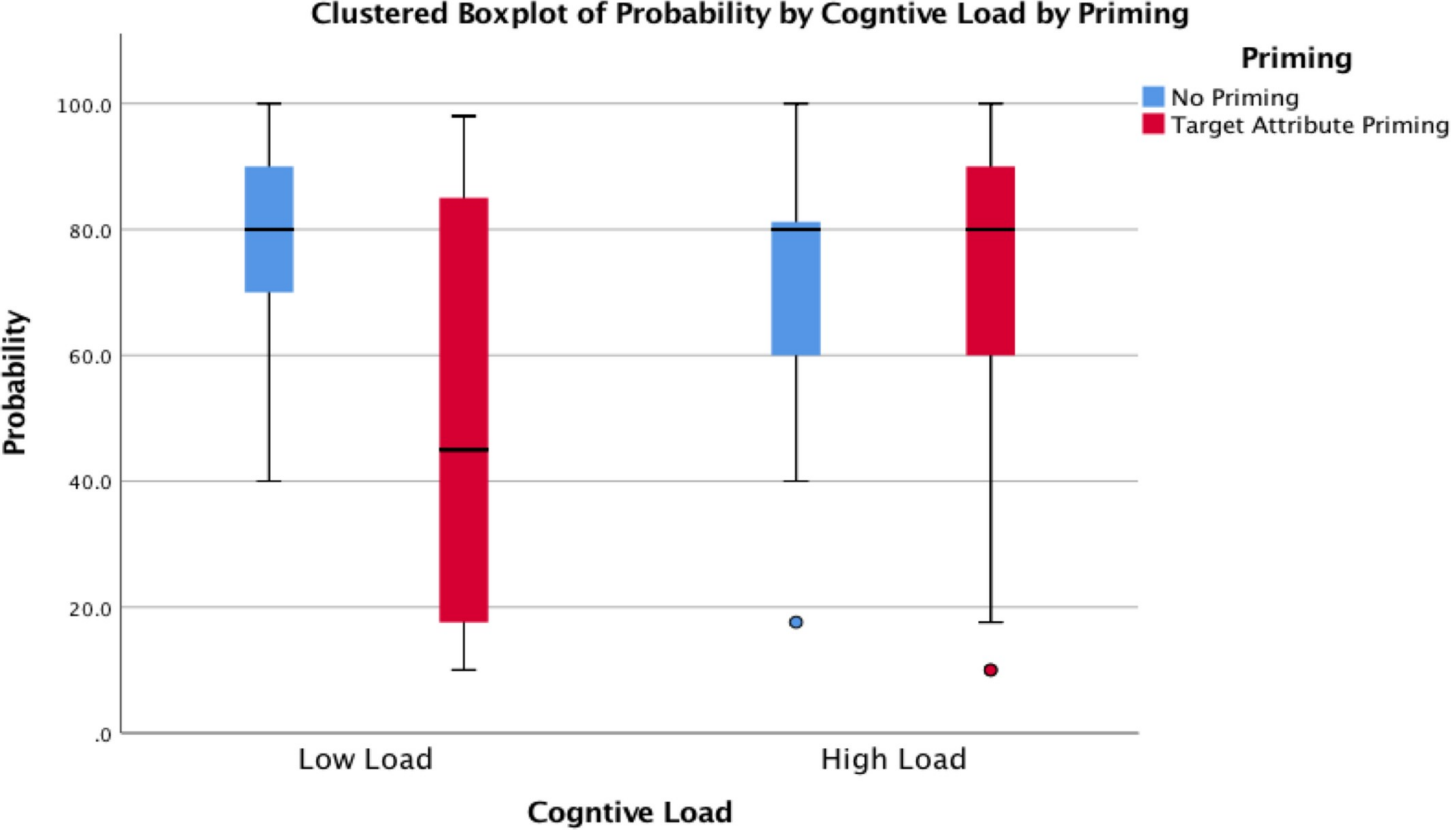
The effect of priming and cognitive load on the base-rate neglect in Experiment 3. Higher numbers indicate greater base-rate neglect.

To analyze the probability estimates, we conducted a 2 x 2 ANOVA. The analysis revealed a main effect of priming (*F*(1, 98) = 10.51, *p* = .002, *η*^*2*^ = .097) and a main effect of cognitive load (*F*(1, 98) = 4.69, *p* = .033, *η*^*2*^ = .046), which is qualified by a significant interaction (*F*(1, 98) = 10.57, *p* = .002, *η*^*2*^ = .097; see Fig 3). In the low cognitive load conditions, participants primed with the target attribute (*M* = 51.24, *SD* = 33.09) were more likely to estimate the probability to be closer to the base rate than those who were not primed (*M* = 79.89, *SD* = 14.22; *t*(52) = 4.08, *p* < .001, *d* = .95). However, in the high cognitive load conditions, no difference was found between the target attribute priming condition (*M* = 75.14, *SD* = 20.35) and the no priming condition (*M* = 75.10, *SD* = 13.96; *t*(46) = -.01, *p* = .994, *d* = .002). Participants’ probability estimates in the high cognitive load conditions were equally far from the base rate, which implies that a reduction in cognitive resources impairs the correction of the base-rate neglect. We also conducted an analysis of the relationship between the number of digits correctly recalled and the probability estimate in the high load condition to examine whether the base-rate neglect increases as the number of digits correctly recalled increases. The regression analysis revealed that the number of digits correctly recalled had a significant positive effect on the probability estimate (*b* = 2.72, *p* = .018). This indicates that increasing cognitive load increases the base-rate neglect, because correctly recalling more digits should require more cognitive resources.

### Base-rate

We also analyzed the percentage of participants who reported the exact base-rate of professors –17.6%. In the low load conditions, 25% of the participants who were primed with the target attribute reported the exact base-rate (i.e., 17.6%), significantly more often than those who were not primed (0%; *χ*^2^(*df* = 1, *N* = 54) = 7.47, *p* = .006, Φ = .37). However, in the high load conditions, very few participants reported 17.6% regardless of whether they were primed with the target attribute (4%) or not (0%; *χ*^2^(*df* = 1, *N* = 48) = 1.10, *p* = .292, Φ = .15). Among the participants who were primed with the target attribute, only 4% of the participants who experienced a cognitive load were able to report the exact base-rate as compared to 25% of the participants who did not experience a cognitive load (*χ*^2^(*df* = 1, *N* = 51) = 4.07, *p* = .044, Φ = -.28).

### PANAS scale

In this analysis, only one hundred and one participants were included since one participant did not complete the PANAS scale. The analysis of the PANAS indicated no differences in positive affect (*M*_*9-digit*_ = 3.90, *SD*_*9-digit*_ = 1.71 vs. *M*_*2-digit*_ *=* 3.76, *SD*_*2-digit*_ = 1.62; *t*(99) = -.41, *p* = .681, *d* = .08) nor any differences in negative affect *(M*_*9-digit*_ = 3.66, *SD*_*9-digit*_ = 2.29 vs. *M*_*2-digit*_ = 3.53, *SD*_*2-digit*_ = 2.37; *t*(99) = -.29, *p* = .775, *d* = .06) as a function of cognitive load, which rules out the mood account.

Experiment 3 provides further evidence that priming the target attribute reduced the bias when participants did not experience cognitive load (low cognitive load conditions). When participants experienced cognitive load (high cognitive load conditions), priming the target attribute did not reduce the bias. These findings suggest that cognitive resources are required to reduce the bias.

One question arises as to the finding that priming the target attribute in the high cognitive load condition did not reduce the judgmental bias. Since both the target attribute and the heuristic attribute are accessible in memory in this condition, individuals may need to decide which one to use. One might argue that the probability estimate in this condition should fall somewhere between the probability estimate in the no priming/high load condition (75%) and the probability estimate in the priming/low load condition (51%). A possible explanation as to why the probability estimate in the target attribute priming/high load condition did not fall somewhere in the middle of the two probability estimates (e.g., 63%) is that reducing cognitive resources may reduce comprehension of the problem. Previous research indicates that the capacity of working memory affects text comprehension [46–48], and that a verbal or counting dual task reduces text comprehension [49]. Since the base rate is provided early in the problem, and the description of the individual is given near the end of the problem, reducing cognitive resources may impair the ability to link the base rate to the description, so increasing the accessibility of target attribute may have not had an effect on reducing the bias.

## General discussion

The goal of the current research was to test a novel indirect debiasing technique for two association-based errors (i.e., the ratio-bias and base neglect). We examined whether increasing the accessibility of the target attribute reduces decision biases. Across three experiments, we consistently find evidence that priming the target attribute reduces two judgmental biases in likelihood estimations. In Experiment 1, participants primed with the target attribute were less likely to show the ratio-bias by choosing the “wrong” option (e.g., 9% tray) less often than those who were not primed. In Experiment 2, we replicated this effect, and showed that participants in the heuristic attribute priming condition did not increase the ratio-bias relative to participants in the no priming condition. In Experiment 3, we found that when participants had sufficient cognitive resources, those who were primed with the target attribute were less likely to exhibit the base-rate neglect than those who were not primed. However, when participants had limited cognitive resources, the priming of the target attribute did not reduce the base-rate neglect.

This research extends the Model of Heuristic Judgment by directly testing it and finds that increasing the accessibility of the target attribute can reduce the likelihood of making these two judgmental biases. Furthermore, this research demonstrates the important role of cognitive resources in correcting these two judgmental biases: the decision maker needs to have enough cognitive resources to reduce the biases in addition to the increased accessibility of the target attribute.

Our findings are qualified by several limitations that suggest fruitful avenues for future research. First, drawing on attribute substitution [16, 17], we proposed that increasing the accessibility of a target attribute by priming the target attribute can reduce these two judgmental biases. Although we showed that priming the target attribute was straightforward and indeed effective in reducing the biases, future research should explore more intensive training interventions to promote enduring reductions in decision biases.

Second, the current research focused on the problems that have a single correct answer in making probability judgments. Future research could examine whether the effect could be extended to the problems that have multiple correct answers [50] and other types of judgmental biases beyond the probability judgments (e.g., anchoring, confirmation bias, hindsight bias).

Third, we examined the effect of priming a target attribute (e.g., probability) on judgmental biases as an indirect way of debiasing. Prior work has shown that training statistical concepts is an effective debiasing method [12, 51, 52]. Future work could compare the effect of priming probability-related concepts with the effect of training people to understand those concepts. Another possible avenue for future research would be to investigate the effect of combining different debiasing techniques. For example, in addition to priming the probability concept, would motivating people to be accurate by providing incentives [15, 48, 53, 54] or training statistical concepts [12, 51, 52] further improve judgmental biases?

Lastly, our work examined the judgmental biases that are mainly cognitive and have financial consequences (e.g., winning a lottery). Previous research has shown that judgmental biases can also have an impact on people’s emotional states. For instance, probability bias (negative social events are extremely likely to occur) has been identified as one of the mechanisms that contribute to social anxiety disorder [55]. Future research could fruitfully study some debiasing techniques to improve people’s emotional well-being. Furthermore, building on prior work linking emotions with decisions [45, 56–61], future research should investigate whether emotions are capable of debiasing judgmental errors.

Our work offers some practical implications for improving decisions. We introduced an indirect debiasing technique by altering the environment rather than altering the decision maker. One way to alter the environment is changing choice architecture where decisions are made. Changing how information is presented can help people better understand decision options and identify good options [62–64]. For instance, presenting nutrition information to consumers in a way that relevant attributes are more salient and noticeable might be helpful in reducing biases and promoting healthy diets.

In conclusion, our work contributes to the literature on judgmental heuristics and biases by introducing a novel debiasing technique. While previous research has focused on direct and explicit methods to enhance judgments (e.g., making people consider the opposite, training statistical concepts and providing the problem in frequency format), across three experiments, we found that priming as an indirect and implicit method involving information about a target attribute can improve judgments.
